# Sex Differences in Morphological and Functional Aspects of Exercise-Induced Cardiac Hypertrophy in a Rat Model

**DOI:** 10.3389/fphys.2019.00889

**Published:** 2019-07-12

**Authors:** Attila Oláh, Csaba Mátyás, Dalma Kellermayer, Mihály Ruppert, Bálint András Barta, Alex Ali Sayour, Marianna Török, Gábor Koncsos, Zoltáng Giricz, Péter Ferdinandy, Béla Merkely, Tamás Radovits

**Affiliations:** ^1^Heart and Vascular Center, Semmelweis University, Budapest, Hungary; ^2^Department of Pharmacology and Pharmacotherapy, Semmelweis University, Budapest, Hungary; ^3^Pharmahungary Group, Szeged, Hungary

**Keywords:** athlete’s heart, exercise-induced hypertrophy, pressure-volume analysis, left ventricular function, sex differences

## Abstract

**Background:** Recent evidences suggest that sex hormones may be involved in the regulation of exercise-induced left ventricular (LV) hypertrophy. However, the sex-specific functional consequences of exercise-induced myocardial hypertrophy is still not investigated in detail. We aimed at understanding the sex-specific functional and morphological alterations in the LV and the underlying molecular changes in a rat model of athlete’s heart.

**Methods:** We divided our young, adult male and female rats into control and exercised groups. Athlete’s heart was induced by a 12-week long swim training. Following the training period, we assessed LV hypertrophy with echocardiography, while pressure-volume analysis was performed to investigate *in vivo* LV function. After *in vivo* experiments, molecular biological studies and histological investigations were performed.

**Results:** Echocardiography and post-mortem measured heart weight data indicated LV hypertrophy in both genders, nevertheless it was more pronounced in females. Despite the more significant relative hypertrophy in females, characteristic functional parameters did not show notable differences between the genders. LV pressure-volume analysis showed increased stroke volume, improved contractility and stroke work and unaltered LV stiffness in both male and female exercised rats, while active relaxation was ameliorated solely in male animals. The induction of Akt signaling was more significant in females compared to males. There was also a characteristic difference in the mitogen-activated protein kinase pathway as suppressed phosphorylation of p44/42 MAPK (Erk) and mTOR was observed in female exercised rats, but not in male ones. Myosin heavy chain α (MHC)/β-MHC ratio did not differ in males, but increased markedly in females.

**Conclusion:** Our results confirm that there is a more pronounced exercise-induced LV hypertrophy in females as compared to the males, however, there are only minor differences regarding LV function. There are characteristic molecular differences between male and female animals, that can explain different degrees of LV hypertrophy.

## Introduction

Physiological cardiac hypertrophy induced by repetitive intense exercise is associated with cardiomyocyte enlargement without sign of fibrotic remodeling or cell damage, that leads to a greater functional reserve to provide improved cardiac performance during the exercise session (Ellison et al., 2012). In contrast to pathological remodeling, this kind of hypertrophy is associated with benefits in myocardial energetic status and functional improvement, resulting in a protective role against cardiovascular diseases (Weiner and Baggish, 2012; Oláh et al., 2016). Although athlete’s heart has been characterized by improved cardiac performance and myocardial enlargement in both genders, recent investigations suggest sex-specific regulation of the development of exercise-induced hypertrophy (Foryst-Ludwig and Kintscher, 2013; Dworatzek et al., 2014).

Most of the studies describe a more pronounced hypertrophic response, a greater relative increase of myocardial mass in female individuals compared to male ones, also in human and experimental animals (Konhilas et al., 2004; Luczak and Leinwand, 2009; Foryst-Ludwig et al., 2011; Dworatzek et al., 2014). Sex-specific molecular regulation of physiological hypertrophy has been reported in studies that were conducted in different animal models of exercise-induced hypertrophy. In previous experimental investigations that used short-term voluntary cage wheel running to train rodents, the molecular differences showed differences in the MAPK system: enhanced proportional increase in Ca^2+^/calmodulin-dependent protein kinase (CaMK) activity and phosphorylation of p38 MAPK and ERK were observed in female animals compared to male counterparts (Konhilas et al., 2004; Dworatzek et al., 2014). The relatively greater hypertrophic response has been shown to be associated with different activation of the protein kinase B (Akt) system and these experimental reports demonstrated that estrogen receptor beta might be directly involved in the sex-specific alterations in exercise-induced hypertrophy (Mahmoodzadeh et al., 2012; Dworatzek et al., 2014). However, in these experimental studies, female sex was associated with increased running performance (distance), which complicates the interpretation.

Although there are obvious sex-specific differences in the molecular aspects of exercise-induced cardiac hypertrophy (EICH), functional consequences are still unclear. There are relatively few studies on female athletes examining cardiac size and function (Pelliccia et al., 1996; Hedman et al., 2015). Comparative non-invasive investigation of athletes could utilize only preload and afterload dependent parameters, those might not describe myocardial mechanics in detail (George et al., 1999; Yilmaz et al., 2013). According to our knowledge, no such direct comparison of exercise-induced functional consequences exists, especially using similar exercise load. Difficulties of such human study include ethical concerns (only the non-invasive possibilities to investigate myocardial mechanics) and it would also require great effort to obtain comparable training workload in male and female elite athletes. Animal models might provide an excellent tool for describing sex-specific differences in the development of exercise-induced hypertrophy, mostly due to the precisely defined exercise training conditions.

Our hypothesis was that similar exercise load results in sex-specific distinction in the degree of hypertrophy, which might be associated with different LV functional consequences in male and female rats. Therefore, we aimed at providing LV functional characterization of exercise-induced hypertrophy in male and female rats, thus providing reliable hemodynamic gender-specific comparisons in a rat model of physiological hypertrophy. Additionally, we investigated sex-specific molecular alterations in exercise-induced LV hypertrophy.

## Materials and Methods

### Animals

This study was carried out in accordance with the principles of the Basel Declaration and recommendations of the Guide for the Care and Use of Laboratory Animals provided by the National Institute of Health (NIH Publication No. 86-23, revised 1996.) and the EU Directive 2010/63/EU. The protocol was approved by the Ethical Committee for Animal Experimentation, Semmelweis University, Budapest (PEI/001/2374-4/2015). All animals received humane care.

Young adult, age-matched, 57–61 days old male (*n* = 24) and female (*n* = 24) Wistar rats were housed in standard rat cages at a constant room temperature (22 ± 2 °C) and humidity with a 12:12-h light-dark cycle. The animals were allowed access to a standard laboratory rat diet and water *ad libitum* during the whole experimental period.

### Experimental Groups

After acclimatization, the rats were divided into four experimental groups: male control (MCo, *n* = 12), male exercised (MEx, *n* = 12), female control (FCo, *n* = 12), and female exercised (FEx, *n* = 12).

### Exercise Training – Rat Model of Physiological Cardiac Hypertrophy

For long-term exercise training, both male and female exercised rats swam for a total period of 12 weeks, for 200 min/day, 5 days a week as previously described (Radovits et al., 2013). For the appropriate adaptation, the duration of swimming was increased 15 min every second training day from a basic 15 min on the first day, until achieving the maximal 200 min/day. The water temperature was maintained at 30–32°C during exercise session. Untrained control rats were placed into the water for 5 min each day during the 12-week training program.

*In vivo* measurements were performed at least 6 h, but not more than 24 h after last exercise session.

### Echocardiography

At the completion of swimming training program, LV morphological alterations were observed by echocardiography using a 13 MHz linear transducer (12L-RS, GE Healthcare, Horten, Norway), connected to a commercially available system (Vivid i, GE Healthcare) as described before (Olah et al., 2017). Rats were anesthetized with pentobarbital sodium (60 mg/kg i.p.). Animals were placed on controlled heating pads, and the core temperature was maintained at 37°C. Standard two-dimensional and M-mode long- and short axis (at mid-papillary level) images were acquired. On two-dimensional recordings of the short-axis at the mid-papillary level, LV anterior (AWT) and posterior (PWT) wall thickness in diastole (index: d) and systole (index: s) as well as LV end-diastolic (LVEDD) and end-systolic diameter (LVESD) were measured. LV volume values (LVEDV and LVESV) were estimated according to the Teichholz’s formula.

Fractional shortening (FS), ejection fraction (EF), and stroke volume (SV) were calculated according to standard formulas. LV mass was determined according to the following formula suggested by Devereux: LVmass = [(LVEDD+AWTd+PWTd)^3^ - LVEDD^3^] × 1.04 (Devereux et al., 1986). To calculate LV mass index, we normalized the LV mass values to the tibial length (TL) of the animal.

### Hemodynamic Measurements – Left Ventricular Pressure-Volume Analysis

After completion of the 12-week long training protocols to induce myocardial hypertrophy, *in vivo* hemodynamic measurements were performed as described previously (Oláh et al., 2016). Shortly, after anesthesia using pentobarbital-sodium (60 mg/kg), using breath and temperature control, a 2-Fr pressure-conductance microcatheter (SPR-838, Millar Instruments, Houston, TX, United States) was inserted into the right carotid artery and advanced into the left ventricle through the ascending aorta.

After stabilization, such as heart rate (HR), LV end-systolic pressure (LVESP), LV end-diastolic pressure (LVEDP), the maximal slope of LV systolic pressure increment (dP/dt_max_) and diastolic pressure decrement (dP/dt_min_), time constant of LV pressure decay [τ; according to Glantz], LV end-diastolic volume (LVEDV), LV end-systolic volume (LVESV), stroke volume (SV), ejection fraction (EF), cardiac output (CO), and stroke work (SW) were calculated and corrected according to *in vitro* and *in vivo* volume calibrations. To exclude the influence of body weight differences, CO was normalized to body weight [cardiac index (CI)].

To obtain load-independent parameters LV P-V relations were measured by transiently compressing the inferior vena cava (reducing preload). The slope of the LV end-systolic P-V relationship (ESPVR; according to the parabolic curvilinear model) and preload recruitable stroke work (PRSW) were calculated as load-independent indices of LV contractility. The slope of the LV end-diastolic PV relationship (EDPVR) was calculated as a reliable indicator of LV stiffness.

Arterial elastance (E_a_) was calculated as LVESP/SV. Ventriculoarterial coupling (VAC) was described by the quotient of E_a_ and ESPVR.

After completing hemodynamic measurements to remove erythrocytes from myocardial tissue, an *in vivo* perfusion was performed. After opening the thoracic cavity and dissecting the inferior caval vein in the thorax, a total volume of 40 ml oxygenated Ringer solution (37°C) was infused into the LV through the apex of the heart. All animals were euthanized by exsanguination.

Thereafter, the heart was quickly removed and placed into cold (4°C) Ringer solution. Heart weight was measured and LV myocardial tissue samples were collected immediately for histology and molecular biology. Subsequently post-mortem TL measurements were done.

### Histology

After organ weight measurements, the hearts were fixed in buffered paraformaldehyde solution (4%) and embedded in paraffin. Transverse, transmural, ∼5 μm thick slices of the ventricles were cut and placed on adhesive slides.

Hematoxylin and eosin staining was performed to measure cardiomyocyte diameter (CD) as a cellular marker of myocardial hypertrophy. In each sample, 100 longitudinally oriented cardiomyocytes from the LV were examined, and the diameters at the transnuclear position were defined. The mean value of 100 measurements represented one sample.

The extent of myocardial fibrosis was assessed on picrosirius-stained sections (Ruppert et al., 2018). ImageJ software (National Institutes of Health, Bethesda, MD, United States) was used to identify the picrosirius-red positive area. Three transmural images (magnification 50 ×) were randomly taken from the free LV wall on each sections. After background subtraction, eye controlled auto-thresholds have been determined to detect positive areas. The fibrosis area (picrosirius red positive area-to-total area ratio) was determined on each image, and the mean value of three images represents each animal.

### Cardiac mRNA Analysis

Left ventricular myocardial tissue samples were harvested immediately after sacrifice, snap-frozen in liquid nitrogen and stored at -80°C. LV tissue of 8-8 animals from each group was homogenized in a lysis buffer (RLT buffer; Qiagen, Hilden, Germany), RNA was isolated from the ventricular samples using the RNeasy Fibrous Tissue Mini Kit (Qiagen) according to the manufacturer’s instructions and quantified by measuring optical density (OD) at 260 nm. RNA purity was ensured by obtaining a 260/280 nm OD ratio approximately 2.0. Reverse transcription reaction (1 μg total RNA of each sample) was completed using the QuantiTect Reverse Transcription Kit (Qiagen). Quantitative real-time PCR was performed with the StepOnePlusTM Real-Time PCR System (Applied Biosystems, Foster City, CA, United States) in triplicates of each sample in a volume of 10 μl in each well containing cDNA (1 μl), TaqMan^®^ Universal PCR MasterMix (5 μl) and a TaqMan^®^ Gene Expression Assay for the following markers (0.5 μl): atrial natriuretic factor (ANF, assay ID: Rn00561661_m1); transforming growth factor β1 (TGF-β, assay ID: Rn00572010_m1) and α and β-isoform of myosin heavy chain (α-MHC, assay ID: Rn00568304_m1; β-MHC, assay ID: Rn00568328_m1) purchased from Applied Biosystems. Gene expression data were normalized to glyceraldehyde-3-phosphate dehydrogenase (GAPDH; reference gene; assay ID: Rn01775763_g1) and expression levels were calculated using the CT comparative method (2^-ΔCT^). All results are expressed as values normalized to a positive calibrator (a pool of cDNA’s from all samples of the control group).

### Western Blot

Western blot experiments were performed as described earlier (Koncsos et al., 2016). Freeze-clamped LV samples from six animals of each group were homogenized with RIPA buffer (Sigma Aldrich, Budapest, Hungary) containing Complete Protease Inhibitor Cocktail (Roche, Basel, Switzerland). Protein concentration was measured by Pierce BCA Protein Assay Kit (Thermo Fisher Scientific, Rockford, IL, United States). Protein concentration was assessed with BCA kit (Thermo Fisher Scientific). Protein samples were resolved on precast 4–20% Criterion TGX gels (Bio-Rad, Hercules, CA, United States) and transferred to Immun-Blot PVDF membranes (Bio-Rad). Equal protein loading was verified with Ponceau staining. Membranes were blocked with bovine serum albumin (BSA; Santa Cruz Biotechnology, Dallas, TX, United States) in Tris-buffered saline with 0.05% Tween 20 (TBS-T) for 2 h. Membranes were incubated with primary antibodies in BSA in TBS-T against phospho-Akt [Ser473] (Catalog number: #4060), Akt (#9272), phospho-p44/42 MAPK (Extracellular signal-regulated kinase, ERK 1/2) [Thr202/Tyr204] (#9101), p44/42 MAPK (ERK 1/2, #9102), phospho-p38 MAPK [Thr180/Tyr182] (#4511), p38 MAPK (#9212), phospho-glycogen synthase kinase (GSK)-3β [Ser9] (#5558), GSK-3β (#12456), phospho-mammalian target of rapamycin (mTOR) [Ser2448] (#2971), mTOR (#2972), phospho-S6 [Ser235/Ser236] (#2211), and S6 (#2217) (all supplied from Cell Signaling). After three washes with TBS-T, horseradish peroxidase-conjugated secondary antibody was added for 1 h at room temperature (in BSA in TBS-T; Cell Signaling). Signals were detected with an enhanced chemiluminescence kit (Bio-Rad) by Chemidoc XRS+ (Bio-Rad) and quantitated in Image Lab 4.1 software (Bio-Rad). Antibodies bound to phospho-epitopes were removed with Pierce Stripping Buffer (Thermo Fisher Scientific) before incubation with antibodies detecting the total protein. We included all intact samples in the analysis.

### Statistics

Results are expressed as mean ± SEM. After confirming normal distribution of data (Shapiro–Wilks method), two-way analysis of variance (ANOVA) with the factors “Sex” and “Exercise” was performed and *p*-values for sex and exercise interaction (p_i_) were calculated. *Post hoc* pairwise comparisons were performed using the Tukey method to determine differences between groups (MCo vs. MEx; FCo vs. FEx). A *p-*value < 0.05 was the criterion of significance.

## Results

### Left Ventricular Hypertrophy

**FIGURE 1:**
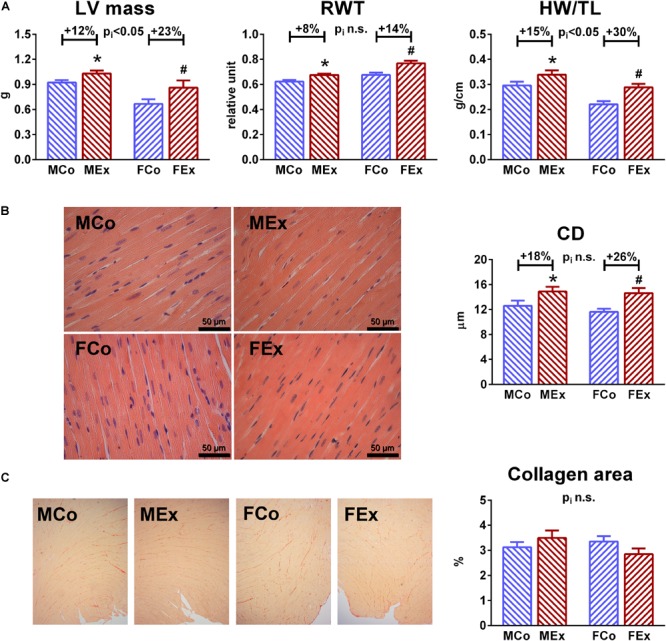
Characterization of exercise-induced left ventricular (LV) hypertrophy. **(A)** Echocardiographic LV mass and post-mortem measured heart weight (HW, normalized to TL) showed increased values in male exercised (MEx) and female-exercised (FEx) animals compared to male control (MCo), and female control (FCo) rats, respectively. Female gender was associated with greater degree of hypertrophy. A slight, but significant increase of relative wall thickness (RWT) suggest concentric type of hypertrophy in our rat model. **(B)** Representative hematoxylin-eosin stained sections (magnification 400 ×) from all of the groups, that were used to measure transnuclear cardiomyocyte width. Mean LV cardiomyocyte diameter (CD) was increased in both genders, that confirmed hypertrophy at cellular level. **(C)** One-one representative picrosirius-stained section from each group. Red color indicates collagen fibers, magnification 50 ×. Picrosirius-staining showed unaltered collagen density in exercised rats nor in male neither in female animals. Values are means ± SEM. ^∗^*p* < 0.05 vs. MCo, ^#^*p* < 0.05 vs. FCo, p_i_: interaction *p*-value of two-way analysis of variance (ANOVA).

Post-mortem measured heart weight and LV wall thickness values, as well as calculated LV mass data by echocardiography indicated cardiac hypertrophy in both exercised groups compared to control ones (Figure 1). There were notable differences between the degree of hypertrophy: female sex was associated with greater relative hypertrophy (Figure 1). We detected concentric hypertrophy in both male and female rats indicated by increased RWT. According to our data, no significant LV dilatation was observed after the completion of swim training program.

Histological analysis also confirmed exercise-induced hypertrophy at the microscopic level, we observed cardiomyocyte enlargement in both exercised groups (Figure 1). Picrosirius staining revealed no collagen deposition in the myocardium of trained rats, suggesting the physiological nature of LV hypertrophy in both genders (Figure 1).

### Left Ventricular Function

**FIGURE 2:**
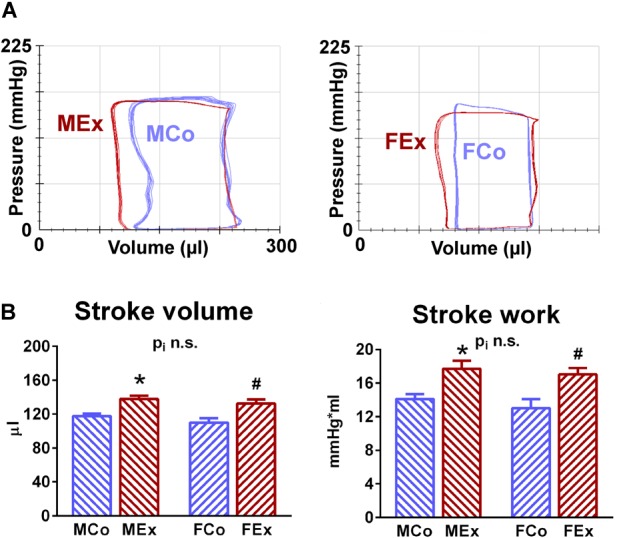
Baseline pressure-volume relations. **(A)** Original recordings of steady-state pressure-volume (P-V) loops obtained from one representative rat from male control (MCo), male exercised (MEx), female control (FCo), and female exercised (FEx) groups. As shown by the recordings, the wider P-V loop indicates increased stroke volume along with unaltered end-diastolic volume and pressure relations, decreased end-systolic volume in exercised rats of both genders compared with untrained controls. **(B)** Exercise-induced hypertrophy was associated with increased stroke volume and stroke work in both genders. Values are means ± SEM. ^∗^*p* < 0.05 vs. MCo, ^#^*p* < 0.05 vs. FCo, p_i_: interaction *p*-value of two-way analysis of variance (ANOVA).

Figure 2 shows illustrative steady-state P-V loops obtained from MCo, MEx, FCo, and FEx animals. The widening of the baseline loops can be observed both in MEx and FEx rats compared to corresponding controls and it clearly reflects increased stroke volume in case of exercise-induced hypertrophy. As the representative P-V loops depict (data shown in Table 2.), exercise training was associated with decreased LVESV along with unaltered LVEDV. These alterations were also confirmed by echocardiographic results (Table 1). Consequently, SV, EF, CI, and SW was increased in exercised rats compared to control ones suggesting increased systolic performance in the hearts, underwent exercise training. We should also mention that neither HR nor pressure relations (MAP, LV pressure values, dP/dt_max_, and dP/dt_min_) did differ between control and exercised groups.

**Table 1:** Left ventricular (LV) echocardiographic data LV echocardiographic data of male control (MCo), male exercised (MEx), female control (FCo), and female exercised (FEx) rats.

	MCo	MEx	FCo	FEx	p_i_
BW, g	468 ± 10	402 ± 11ˆ*	291 ± 8	281 ± 7	0.004
HR, 1/min	355 ± 14	347 ± 11	362 ± 9	342 ± 8	0.580
LVAWTd, mm	2.15 ± 0.02	2.37 ± 0.03ˆ*	2.03 ± 0.04	2.37 ± 0.04ˆ#	0.085
LVAWTs, mm	3.13 ± 0.08	3.52 ± 0.06ˆ*	3.09 ± 0.06	3.58 ± 0.10ˆ#	0.536
LVPWTd, mm	1.96 ± 0.03	2.10 ± 0.02ˆ*	1.73 ± 0.02	1.96 ± 0.04ˆ#	0.145
LVPWTs, mm	2.97 ± 0.06	3.16 ± 0.07ˆ*	2.77 ± 0.04	3.10 ± 0.04ˆ#	0.198
LVEDD, mm	6.57 ± 0.08	6.63 ± 0.06	5.59 ± 0.12	5.72 ± 0.09	0.700
LVESD, mm	3.91 ± 0.10	3.26 ± 0.11ˆ*	3.12 ± 0.09	2.71 ± 0.09ˆ#	0.227
FS, %	40.6 ± 1.2	50.9 ± 1.3ˆ*	44.2 ± 0.8	52.6 ± 1.3ˆ#	0.430
LVEDV, μl	221.8 ± 5.8	226.3 ± 4.9	153.6 ± 7.5	161.7 ± 6.0	0.771
LVESV, μl	66.8 ± 3.8	43.6 ± 3.3ˆ*	39.0 ± 2.8	27.8 ± 2.0ˆ#	0.054
SV, μl	155.0 ± 4.6	182.8 ± 3.0ˆ*	114.6 ± 5.4	133.9 ± 5.2ˆ#	0.375
EF, %	70.0 ± 1.4	80.9 ± 1.2ˆ*	74.7 ± 0.9	82.8 ± 1.1ˆ#	0.242

*Values are means ± SEM. BW, body weight; HR, heart rate; LVAWTd and LVAWTs, left ventricular (LV) anterior wall thickness at diastole and systole, respectively; LVPWTd and LVPWTs, LV posterior wall thickness at diastole and systole, respectively; LVEDD, LV end-diastolic dimension; LVESD LV end-systolic dimension; FS, fractional shortening; LVEDV, LV end-diastolic volume; LVESV, LV end-systolic volume; SV, stroke volume; EF, ejection fraction. ^∗^*p* < 0.05 vs. MCo, ^#^*p* < 0.05 vs. FCo. p_*i*_: interaction value (factors: gender and exercise) of two-way analysis of variance (ANOVA).*

**FIGURE 3:**
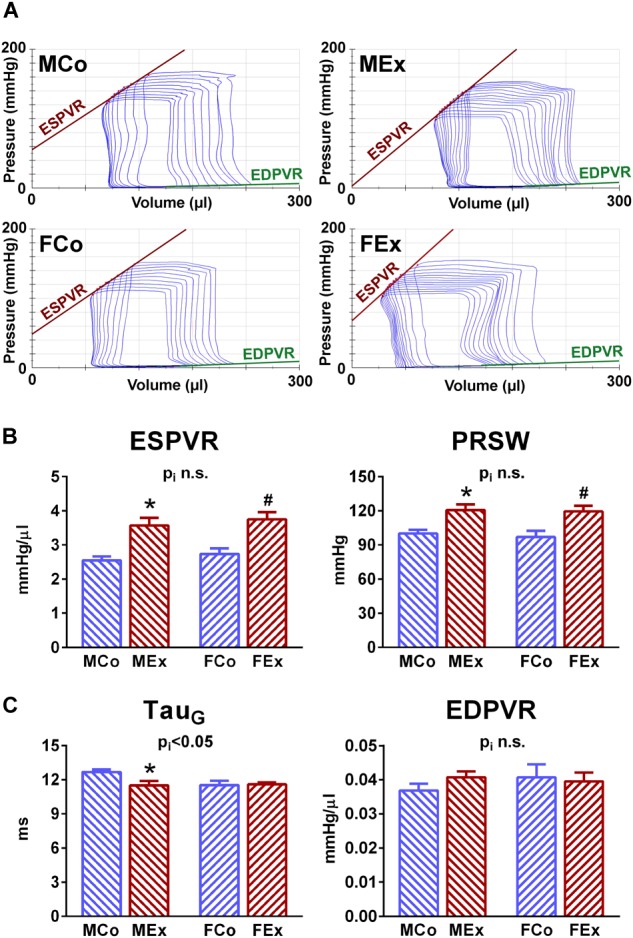
Load-independent indices of LV systolic and diastolic function. **(A)** Characteristic indices of LV contractility and stiffness could be obtained by pressure-volume (P-V) analysis during reducing cardiac preload by the transient occlusion of vena cava inferior. Representative original recordings of P-V relations from one animal of each group during this maneuver were depicted. **(B)** Sensitive contractility indices, such as the slope of end-systolic pressure-volume relationship (ESPVR) and PRSW revealed comparable exercise-induced LV contraction improvement in both genders. **(C)** There was a notable difference regarding Tau, a load-independent parameter of LV active relaxation. In male rats a diastolic improvement was shown as indicated by decreased Tau values, whereas exercise was not associated with altered Tau in females. The slope of end-diastolic pressure volume relationship (EDPVR), that describes LV stiffness, has not been altered by exercise training. MCo, male control group; MEx, male exercised group; FCo, female control group; FEx, female exercised group. Values are means ± SEM. ^∗^*p* < 0.05 vs. MCo, ^#^*p* < 0.05 vs. FCo, p_i_: interaction *p*-value of two-way analysis of variance (ANOVA).

To obtain load-independent, sensitive functional parameters we recorded P-V relations while cardiac preload was altered. Figure 3 displays representative P-V loops obtained during inferior vena cava occlusions in MCo, MEx, FCo, and FEx rats. Overall results of ESPVR and PRSW have been shown on Figure 3. Both contractility parameters were increased in both genders at a similar degree.

τ, that is a load-independent parameter of LV relaxation, a major determinant of diastolic function, was significantly shortened (thus improved) in the case of male exercised animals, whereas there was no alteration in female rats compared to their control counterparts (Figure 3).

We also determined EDPVR, which characterizes LV stiffness. Although marked hypertrophy was observed in both genders, this parameter was not altered neither in male nor in female animals (Figure 3).

**FIGURE 4:**
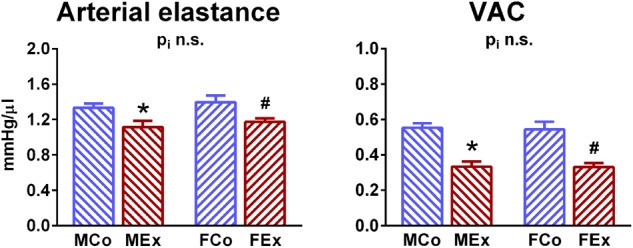
Comparison of ventriculo-arterial connection. Arterial elastance was decreased in both genders and ventriculo-arterial coupling (VAC) was improved in both genders. MCo, male control group; MEx, male exercised group; FCo, female control group; FEx, female exercised group. Values are means ± SEM. ^∗^*p* < 0.05 vs. MCo, ^#^*p* < 0.05 vs. FCo, p_i_: interaction *p*-value of two-way analysis of variance (ANOVA).

We also investigated parameters that describe the connection of ventricular and arterial system (Figure 4). Arterial elastance has been found to be decreased in both genders: increased stroke volume was detected along with unchanged LV pressure values in exercised rats. Additionally, VAC showed a more optimized ventriculo-arterial interaction in exercised rats compared to controls.

### Molecular Differences

**FIGURE 5:**
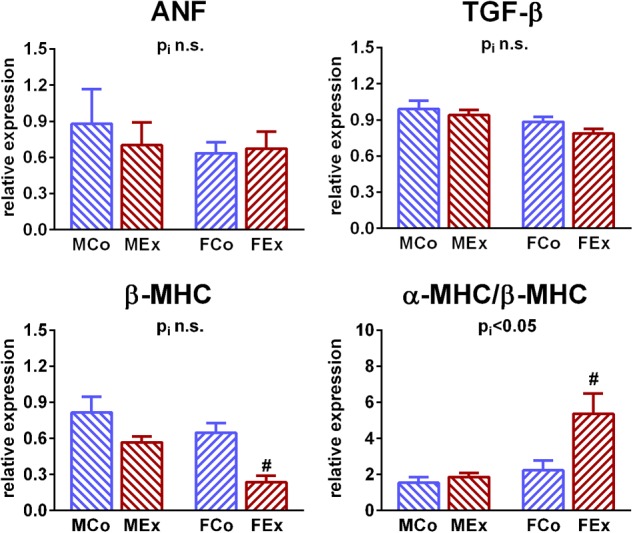
Left ventricular gene expression analysis. LV gene expression of atrial natriuretic factor (ANF) and transforming growth factor beta (TGF-β) did not alter in exercised animals. Expression of β-myosin heavy chain (MHC) mRNA was decreased in exercised female animals, that resulted in an increase of α-MHC/β-MHC ratio, while there was no difference between male exercised and control rats. MCo, male control group; MEx, male exercised group; FCo, female control group; FEx, female exercised group. Values are means ± SEM. ^∗^*p* < 0.05 vs. MCo, ^#^*p* < 0.05 vs. FCo, p_i_: interaction *p*-value of two-way analysis of variance (ANOVA).

Left ventricular gene expression values of markers that are associated with pathological remodeling of the left ventricle (ANF, TGF-β) did not differ between the groups. There were characteristic differences between male and female rats in the mRNA expression of myosin heavy chain types. We detected a decreased expression of β-MHC in exercised female animals compared to control ones, that resulted in a marked increase of α-MHC/β-MHC ratio, while there was no difference between male exercised and control rats (Figure 5).

**FIGURE 6:**
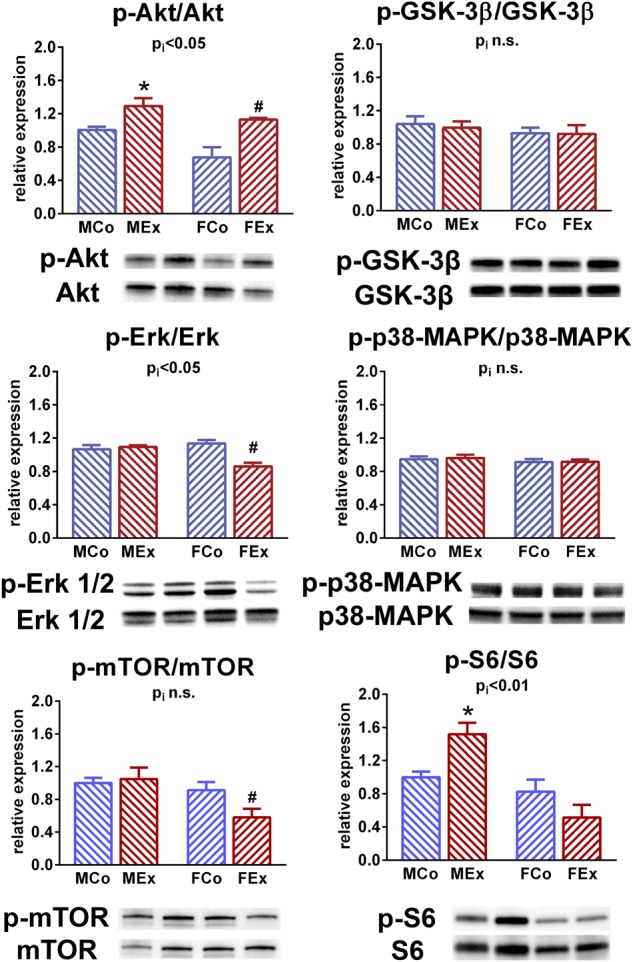
Western blot. Protein kinase B (Akt) phosphorylation was increased in male and female trained animals in comparison with control counterparts. This alteration was more pronounced in exercised female exercised rats compared to male ones. We have not found any differences regarding the phosphorylation of p38 mitogen activated protein kinase (MAPK) and glycogen synthase kinase (GSK)-3β. Extracellular signal-regulated kinase (Erk) 1/2 (p42/44 MAPK) phosphorylation was decreased in female animals, and remained unaltered in male rats after the training program. Phosphorylation of mammalian target of rapamycin (mTOR) showed exercise-induced alteration only in female animals, while phosphorylation of S6 ribosomal protein was increased only in male exercised animals. MCo, male control group; MEx, male exercised group; FCo, female control group; FEx, female exercised group. Values are means ± SEM. ^∗^*p* < 0.05 vs. MCo, ^#^*p* < 0.05 vs. FCo, p_i_: interaction *p*-value of two-way analysis of variance (ANOVA).

We performed additional molecular biological measurements to investigate underlying molecular mechanisms behind the differences in exercise-induced hypertrophy between sexes. Phosphorylation of Akt was increased in exercised male and female animals as compared to control counterparts, however, it was relatively more pronounced in female as in male rats. Exercise or sex had no effect on the phosphorylation of p38 MAPK and GSK-3β. Phosphorylation of Erk1/2 (p42/44 MAPK) was unaltered in exercised male rats compared to control ones, however, it was decreased by training in female animals (Figure 6).

The phosphorylation of mTOR was decreased in female exercised animals compared to their controls, while exercise training did not alter mTOR-phosphorylation in male rats. We found sex-related distinction in the phosphorylation of S6 ribosomal protein: exercise was associated with an increment of S6 phosphorylation in male animals, while it did not differ between female control and exercised rats (Figure 6).

## Discussion

We provided the first detailed *in vivo* hemodynamic comparison of exercise-induced hypertrophy in male and female rats. Our exercised animals of both genders underwent an identical swim training program (similar workload) to enable a reliable comparison of LV functional consequences of long-term exercise training.

### Left Ventricular Hypertrophy

Numerous human studies and experimental examinations investigated the sex-dependency of hypertrophic response induced by exercise training. However, it might be challenging to obtain similar workload in male and female highly elite athletes, thus human data about the sex-specific response of exercise-induced hypertrophy are controversial (Pelliccia et al., 1991; Yilmaz et al., 2013; Finocchiaro et al., 2017). Experimental studies were frequently using voluntary cage wheel running systems, that might also lead to different workload, because female mice tend to run much longer and faster voluntarily than males (Luczak and Leinwand, 2009). Our results clearly indicate a relatively more pronounced cardiac and left ventricular (LV) hypertrophy in female animals compared to male ones after completing the 12-week long training program, while we detected ∼15–20% relative increase of cardiac mass in male animals, female exercised rats were related to a ∼25–30% growth of myocardial tissue (Figure 1). These alterations are in line with most of the conducted studies in rodents, using voluntary or forced training systems (Konhilas et al., 2004; Foryst-Ludwig et al., 2011; Dworatzek et al., 2014). Histological analysis revealed alterations that are characteristic to physiological hypertrophy (Bernardo et al., 2010; Ellison et al., 2012; Oláh et al., 2016): cardiomyocyte enlargement without myocardial fibrosis. The unaltered ANF and TGF-β values (Figure 5) also confirmed the physiological nature of the observed hypertrophy (Bernardo et al., 2010; Oláh et al., 2016). Moreover, β-MHC, another pathological hypertrophy marker has not been only unaltered in female animals, but exercise training resulted in decreased myocardial expression, that also leaded to increased α/β-MHC values. Indeed, there are evidences that myocardial expression of β-MHC shows sex-specific differences also in pathological conditions (Rosenkranz-Weiss et al., 1994; Zhong et al., 2003).

We found concentric type of hypertrophy in our rat model of exercise-induced hypertrophy according to increased LV wall thickness and RWT values and unaltered end-diastolic dimensions measured by echocardiography and pressure-volume analysis (Figure 1 and Tables 1, 2). According to the Morganroth hypothesis and recent investigations in athletes, regular aerobic exercise training – such as swim training – induces rather an eccentric type of LV hypertrophy (Naylor et al., 2008). The dilatation of LV in diastole might be related to exercise-induced resting bradycardia, because of relatively prolonged LV filling and elongated diastole during the heart cycle (Pavlik et al., 2013). Hence, we should mention that during our *in vivo* measurements, animals were anesthetized, and in that condition we could not detect alterations in HR values (Tables 1, 2), which can be explained by the impact of anesthesia on the autonomic nervous system. However, this discrepancy between our results and human studies might not influence parameters of pressure-volume analysis, that are independent of cardiac load and HR (Pacher et al., 2008).

**Table 2:** Left ventricular hemodynamic data LV hemodynamic data of male control (MCo), male exercised (MEx), female control (FCo), and female exercised (FEx) rats.

	MCo	MEx	FCo	FEx	p_i_
HR, 1/min	414 ± 9	400 ± 8	400 ± 12	397 ± 8	0.562
MAP, mmHg	140.0 ± 5.1	138.3 ± 6.1	138.2 ± 5.0	142.2 ± 4.1	0.582
LVESP, mmHg	156.5 ± 3.9	152.3 ± 8.2	148.7 ± 6.2	155.5 ± 5.7	0.380
LVEDP, mmHg	3.3 ± 0.7	4.1 ± 1.5	3.7 ± 1.1	4.1 ± 1.1	0.861
dP/dt_max_, mmHg/s	9241 ± 397	9821 ± 667	9331 ± 565	10231 ± 654	0.785
dP/dt_min_, mmHg/s	–12246 ± 432	–12211 ± 670	–12579 ± 655	–13285 ± 487	0.521
LVEDV, μl	229.9 ± 2.9	239.4 ± 4.6	210.1 ± 4.3	216.1 ± 5.2	0.689
LVESV, μl	112.2 ± 1.8	101.6 ± 1.8ˆ*	100.3 ± 4.0	83.8 ± 2.7ˆ#	0.287
EF, %	51.5 ± 1.1	57.5 ± 0.7ˆ*	52.7 ± 1.9	61.1 ± 1.3ˆ#	0.370

*Values are means ± SEM. HR, heart rate; MAP, mean arterial pressure; LVESP, LV end-systolic pressure; LVEDP, LV end-diastolic pressure; dP/dt_max_, maximal slope of the systolic pressure increment; dP/dt_min_, maximal slope of the diastolic pressure decrement; LVEDV, LV end-diastolic volume; LVESV, LV end-systolic volume; EF, ejection fraction. ^∗^*p* < 0.05 vs. MCo, ^#^*p* < 0.05 vs. FCo. p_*i*_: interaction value (factors: gender and exercise) of two-way analysis of variance (ANOVA).*

We also investigated hypertrophy-associated molecular pathways (Figure 6). Akt, a serine-threonine kinase as the main effector of physiological hypertrophy-associated IGF-1/PI3K/Akt pathway plays a pivotal role in the development of EICH (Bernardo et al., 2010; DeBosch et al., 2006). The active, phosphorylated form of Akt was increased in both male and female animals, that is in line with other studies that investigated physiological hypertrophy (Weeks et al., 2017). In female rats, Akt activation seemed to be more pronounced, which is in line with the relatively increased hypertrophic response in female rats compared to alterations in male animals (Figure 4).

Glycogen synthase kinase-3β, a cellular substrate for Akt, is an important regulator with anti-hypertrophic effect and inhibition of GSK-3β has been proposed as an important mechanism for stimulating growth in hypertrophy (Antos et al., 2002; Bernardo et al., 2010). According to our data, phosphorylation of GSK-3β did not differ between the exercised and control groups after 12 weeks of swim training, which might suggest that the active development process of hypertrophy was completed (Figure 6). This is in line with a study, where GSK-3β phosphorylation was altered in the early phase of training, while there was no alteration detected later (Konhilas et al., 2004). The activation of mTOR signal pathway and its downstream substrates have also been associated with the development of exercise-induced hypertrophy (Kemi et al., 2008). We found sex-related differences in mTOR phosphorylation and S6 ribosomal protein phosphorylation (Figure 6), that suggest a distinct regulation of mTOR pathway in male and female animals.

Although their role in the development of physiological hypertrophy is still doubtful, we also investigated proteins of MAPK system that are proposed for sex-specific exercise-induced effects and are hypothetically related to estrogen receptors (Regitz-Zagrosek et al., 2010; Dworatzek et al., 2014). p38-MAPK has been implicated in the regulation of cardiac gene expression, cardiac myocyte apoptosis, myocyte hypertrophy, contractility, remodeling and metabolism. We have not found any differences regarding activation of p38-MAPK, which is in line with an investigation of exercise-induced hypertrophy (Konhilas et al., 2004), however, it contradicts another report showing the pivotal role in sex-specific regulation (Dworatzek et al., 2014). Another important MAPK is ERK1/2 (p42-44-MAPK) that is clearly involved in the development of pathological hypertrophy, however, the over activation of ERK was not observed in the case of physiological stimuli (Clerk et al., 2006). We found a decreased phosphorylation of ERK 1/2 in hearts of female exercised rats, while the activation was unaltered in male animals (Figure 5).

It is indeed difficult to interpret results about activation of proteins involved in hypertrophic response, because their phosphorylation might be altered during a different phase of the development of exercise-induced hypertrophy, thus further experimental investigations are needed using standard training protocols to describe activation at different phases during the development of hypertrophy.

### Left Ventricular Function

There are only a few studies in human, that compare exercise-mediated cardiac hypertrophy between male and female athletes, while most of the studies have been focused on male individuals. Although, we have significant data about gender differences in morphological aspect of exercise-induced hypertrophy, the sex-specific functional consequences are still unclear.

#### Systolic Function

Improved systolic performance of athlete’s heart is a typical feature, however, the clinical evaluation of this improvement is yet to be resolved. Increased stroke volume is a characteristic alteration in athlete’s heart in both genders (Yilmaz et al., 2013), that was also confirmed by our echocardiographic and hemodynamic data (Table 1 and Figure 3). We also detected increased FS and EF in our exercised animals. Conventional parameters of systolic function, including LV FS and EF, are unreliable in athletes, while there are obvious alterations in cardiac preload and HR.

Although speckle-tracking echocardiography is a promising tool to follow-up systolic improvement (Kovacs et al., 2015), LV contractility can be assessed precisely and reliably by pressure-volume analysis. Pressure-volume recordings during a transient preload reduction maneuver (vena cava inferior occlusion) provide the opportunity to calculate load-independent indicators of ventricular contractility (Pacher et al., 2008). The most widely used sensitive contractility indices, ESPVR and PRSW, were significantly elevated in trained animals independently from sex (Figure 4). The exercise-mediated improvement in contractility was comparable in male and female rats.

#### Diastolic Function

There are two main components that describe diastolic performance of the ventricular tissue: active relaxation and myocardial stiffness.

Left ventricular relaxation has been identified as an active, energy consuming process and depends mostly on calcium reuptake by the sarcoplasmic reticulum during the early diastole. The time constant of LV pressure decay has been described as a relatively load-independent index of LV active relaxation, and the prolongation of this parameter has been widely described in pathological cardiac conditions (Zhao et al., 2008). Physiological hypertrophy was associated with enhanced relaxation in exercised male animals compared to control ones, as shown by decreased τ (Figure 3). In contrast, no alteration was observed between female control and exercised animals. According to our data, there is a sex-specific difference between male and female animals, that disappears after exercise training (Figure 3), and sexual dimorphism regarding diastolic function has indeed been reported (Luczak and Leinwand, 2009). Active relaxation is a determinative parameter and its characteristic alterations can differentiate between physiological and pathological cardiac diseases. Further investigation of active relaxation might answer the question whether lower female active relaxation time would be protective against diastolic dysfunction, and its improvement would provide additional cardiac performance improvement in male athletes.

Another important marker of diastolic function, describing pressure alterations during ventricular filling, is ventricular stiffness. This feature is affected predominantly by collagen metabolism in the extracellular matrix (ECM), but alterations in myocardial intracellular and other extracellular structural components (e.g., fibrosis) can also influence it. EDPVR, a sensitive marker of LV stiffness, was not altered by exercise training and showed similarity between genders (Figure 3). This result is in good agreement with the absence of marked collagen deposition observed in the hearts of exercised animals (Figure 1) and with our previous findings in physiological hypertrophy (Oláh et al., 2016).

#### Ventricular Mechanics

Long-term exercise training was associated with increased stroke work in both sexes, reflecting improved effective external mechanical work of LV. Determining oxygen consumption in these animals would add further information about the efficiency of LV.

#### Ventriculo-Arterial Interaction

As a feature of the interaction between heart and arterial system, ventricular-arterial coupling expresses the interaction between left ventricle and arterial system using the ratio of arterial elastance and LV end-systolic elastance (Sunagawa et al., 1983). Arterial elastance, an integrative index that includes among others peripheral vascular resistance, arterial compliance and characteristic impedance. Decreased arterial elastance in exercised rats, independently from sex, reveals a better compliance of the arterial system in the case of exercise-induced cardiovascular alterations (Figure 4). Improved VAC in exercise-trained animals reflects a more appropriate matching between the LV and the arterial system and suggests that endurance-trained individuals are able to match peripheral vascular changes with changes in the LV function following dynamic exercise. Optimized ventriculo-arterial coupling has been described only in male athletes, however, this is the first report to provide it in female individuals (Florescu et al., 2010).

## Conclusion

In conclusion, we provided the first detailed hemodynamic comparison of physiological hypertrophy in a rodent model of athlete’s heart. We confirmed physiological cardiac hypertrophy in both genders, which was more pronounced in female animals. We found that activation of Akt was increased in both genders, but even more in female rats and there were gender differences regarding ERK1/2, mTOR and S6 activation and αα/ββ-MHC proportion. Despite the differences in the degree of hypertrophy, only minor differences have been detected during functional measurements.

Both male and female hearts were associated with improved left ventricular contractility, similar “supernormal” systolic function. Differences were detected in early diastolic function: active relaxation was improved solely in male animals. LV stiffness was not affected by exercise training. LV mechanics improved by a comparable degree in the heart of male and female rats. A more optimized ventriculo-arterial interaction (VAC) is also a characteristic feature of both genders.

### Limitations

The interpretation of results from the current study is limited to young male rats. The possible influence of age should be assessed in future studies. Furthermore, the present study was specifically designed to investigate sex differences in the functional consequences in a relevant model of exercise-induced hypertrophy. Also, the length of training period as well as type and intensity of training sessions might affect the observed phenotype.

*In vivo* investigations (echocardiography, pressure-volume analysis) could be performed under anesthesia, which might have an influence on parameters dependent on the autonomic nervous system, such as HR and pressure values. Even so, pressure-volume analysis might provide parameters that are independent of HR and loading conditions.

## Ethics Statement

This study was carried out in accordance with the principles of the Basel Declaration and recommendations of the Guide for the Care and Use of Laboratory Animals provided by the National Institutes of Health (NIH Publication No. 86-23, revised 1996) and the EU Directive 2010/63/EU. The protocol was approved by the Ethical Committee of Hungary for Animal Experimentation. All animals received humane care.

## Author Contributions

AO, BM, and TR conceived or designed the work. AO, CM, DK, MR, BB, AS, MT, GK, and TR acquired the data. AO, CM, DK, GK, ZG, and TR analyzed and interpreted the data. AO and TR were significant manuscript writers and drafted the work. ZG, PF, BM, and TR significantly revised the manuscript. All authors have read the manuscript and provided approval for its submission to Frontiers in Physiology and agreed to be accountable for all aspects of the work in ensuring that questions related to the accuracy or integrity of any part of the work are appropriately investigated and resolved.

## Conflict of Interest Statement

The authors declare that the research was conducted in the absence of any commercial or financial relationships that could be construed as a potential conflict of interest.
